# The silent danger of social distancing

**DOI:** 10.1017/S0033291720002597

**Published:** 2020-07-06

**Authors:** André Aleman, Iris Sommer

**Affiliations:** Department of Biomedical Sciences of Cells and Systems, University of Groningen, University Medical Center Groningen, Netherlands

Human beings have a strong need to connect to other human beings. This social inclination is part and parcel of the *condition humaine*. Michiko Kakutani (2015), in a review of Jonathan Franzen's (https://www.nytimes.com/2015/08/25/books/review-in-purity-jonathan-franzen-hits-a-new-octave.html) book ‘Purity’ published in the *New York Times*, praised his ability to ‘capture his characters’ yearnings for connection’. Apparently, such yearning for connection strikes a universal chord. At the same time, the fact that it takes a star-writer to put it into words, indicates this phenomenon is not an easily observable and readily describable behavior, but rather a deeply felt private emotion that may not be easily accessible to others.

This need for social connections supports cooperation and thereby survival and may explain why we have cultures. Such connections foster social affiliative behaviors (e.g. altruism, cooperation), which are vital for utterly dependent offspring to reach the reproductive age. The same is true for the very old and diseased, who also rely on care of others. Social connectedness improve our chances of survival in difficult or hostile environments.

Many people in modern society feel lonely, with numbers ranging from 20% to 45% of the population in western countries, depending on the way of scoring (Cacioppo, Grippo, London, Goossens, & Cacioppo, 2015). Loneliness has been defined in scientific literature as *perceived social isolation*. Definitions of loneliness emphasize that it involves a discrepancy between an individual's preferred and actual social relationships, which leads to the negative experience of feeling alone. Thus, loneliness is regarded as a distressing feeling that accompanies the perception that one's social needs are not being met by the quantity or especially the quality of one's social relationships (Hawkley & Cacioppo, 2010).

The Covid-19 pandemic has urged most countries to impose rules for social distancing to degrees that vary per country and per region. In areas with high infection rates, such as Northern Italy, the larger Madrid area and Brussels, citizens were prohibited to leave their house for some two to three months, with a short shopping tour being the only exception. Other countries, such as Australia, Germany, the United States and Brazil, were less strict, yet also cancelled all social events and sports, urged people to work from home wherever possible and largely limited possibilities for visiting friends and family. While these rules are applied to preserve public health, they have detrimental effects for quality of life and wellbeing, including mental and physical health (McGinty, Presskreischer, Han, & Barry, 2020). For individuals living alone and those who otherwise have limited social networks, contact with colleagues at work or with team members at sport activities are of vital importance.

Indeed, loneliness is not only an unpleasant state of being, but also has significant health implications. Higher premature mortality has consistently been documented for people with perceived social isolation (Holt-Lunstad, Smith, Baker, Harris, & Stephenson, 2015). Across studies, the weighted average effect sizes, expressed as odds ratio were 1.29 for social isolation, 1.26 for loneliness and 1.32 for living alone, corresponding to an average of 29%, 26% and 32% increased likelihood of death, respectively. These effect sizes are comparable in size to those of well-known risk factors such as hypertension, hyperglycemia and hypercholesterolemia. Unsurprisingly, loneliness also affects psychological well-being, inducing lower self-esteem, higher anger, higher fear of negative evaluation, lower optimism, lower positive mood and higher negative mood (Hawkley & Cacioppo, 2010). Such adverse effects of loneliness may be most pronounced in elderly and diseased subjects, were loneliness is already highest (Altschul, Iveson, & Deary, 2020). In the elderly, loneliness is a robust predictor of cognitive decline with aging and ultimately dementia (Montoliu, Hidalgo, & Salvador, 2019). This is of additional concern, as these are exactly the groups to whom the strictest rules for social distancing are applied during the Covid-19 pandemic.

Hence, social distancing during the Covid-19 pandemic is a two-edged sword; it lowers the risk of infection, but at the same time, increases loneliness, with opposite effects on morbidity and mortality. Whether mortality from Covid-19 infection outweighs mortality associated with loneliness, depends not only on the local degree of virus infection, but also on the length and degree of social isolation measures. For policy makers this is a difficult consideration. We can provide some recommendations here. For example, when prioritizing people allowed to return to work, not only workers in essential professions (e.g. food production, health care), but also professionals living alone should be considered as high priority, to soften the effects of social isolation in this vulnerable group. In addition, prevention of loneliness should be part of general health policy in these times of social distancing. Gardiner, Geldenhuys, and Gott (2018) identified several effective interventions to reduce loneliness including befriending, development of leisure time skills, health or social care interventions, pet programs, psychotherapy and social facilitation programs. Interestingly, solitary interventions involving video conferencing or providing pets, were also effective, which are feasible solutions even during strict quarantine. For example, a study investigated efficacy of an internet training intervention for community-dwelling older people who lived alone, who were housebound through chronic illness or physical disability At the three-year follow-up, intervention group participants reported a significantly greater reduction in overall loneliness in comparison with the control group. This supports the strategy proposed by the Commonwealth Fund, to screen for social isolation and foster digital support by expanding access to telehealth (https://www.commonwealthfund.org/blog/2020/how-covid-19-pandemic-could-increase-social-isolation-and-how-providers-and-policymakers).

In conclusion, social distancing to avoid further spreading of Covid-19 comes with a price. Loneliness reduces quality of life and increases mortality; hence, social distancing rules should be kept as short as possible. Individuals living alone should be considered a priority group to return to work. Finding flexible ways to allow certain forms of face-to-face interaction, e.g. meeting relatives outside and with 1.5 m distance, will reduce risks of loneliness and its adverse effects. Ultimately, preventive strategies to reduce loneliness will benefit those concerned significantly, both in terms of somatic health as well as mental health.
